# Environmentally Relevant Concentrations of Triphenyl Phosphate (TPhP) Impact Development in Zebrafish

**DOI:** 10.3390/toxics12050368

**Published:** 2024-05-16

**Authors:** Benjamin Schmandt, Mfon Diduff, Gabrielle Smart, Larissa M. Williams

**Affiliations:** Biology Department, Bates College, Lewiston, ME 04240, USA

**Keywords:** triphenyl phosphate, zebrafish, cardiotoxicity, tbx5, natriuretic peptides

## Abstract

A common flame-retardant and plasticizer, triphenyl phosphate (TPhP) is an aryl phosphate ester found in many aquatic environments at nM concentrations. Yet, most studies interrogating its toxicity have used µM concentrations. In this study, we used the model organism zebrafish (*Danio rerio*) to uncover the developmental impact of nM exposures to TPhP at the phenotypic and molecular levels. At concentrations of 1.5–15 nM (0.5 µg/L–5 µg/L), chronically dosed 5dpf larvae were shorter in length and had pericardial edema phenotypes that had been previously reported for exposures in the µM range. Cardiotoxicity was observed but did not present as cardiac looping defects as previously reported for µM concentrations. The RXR pathway does not seem to be involved at nM concentrations, but the *tbx5a* transcription factor cascade including natriuretic peptides (*nppa* and *nppb*) and bone morphogenetic protein 4 (*bmp4*) were dysregulated and could be contributing to the cardiac phenotypes. We also demonstrate that TPhP is a weak pro-oxidant, as it increases the oxidative stress response within hours of exposure. Overall, our data indicate that TPhP can affect animal development at environmentally relevant concentrations and its mode of action involves multiple pathways.

## 1. Introduction

Triphenyl phosphate, also known as TPhP or TPP, is a common organophosphorus flame-retardant (OPFR) plastic additive. Its use became popular as a replacement for brominated flame retardants (BFRs), such as hexabromocyclododecane, which were banned due to their persistence, bioaccumulation, long range transport, and adverse effects [1,2,3,4]. Frequently included in electronics, car seats, and nail polish, TPhP has seen increased use in the past two decades due to its unusually short environmental degradation time relative to other OPFRs; it breaks down quickly in the environment, with a half-life of 3–12 days in water and sediment and 30–60 days in anoxic environments [5]. However, as a plastic additive that is not bound to a polymer material, TPhP has high potential to leach from plastics and is often detected in house dust and surface freshwater [6,7].

Studies of tap water as well as lakes and rivers frequently report TPhP in the range of a few to hundreds of ng/L [8,9,10]. Levels of TPhP in polluted areas—such near industrial plants or heavily urbanized zones—have been regularly measured in the range of 0.01–1 µg/L, with record-setting environmental levels of 7.8 µg/L in river water in Denmark [11,12]. Reports on the ability of TPhP to bioaccumulate have been variable because it is readily metabolized and has a lower affinity for lipids than other OPFRs [13]; reported bioconcentration factors in fish range from 0.6 to 1743 [14,15,16]. The very wide range in these values indicates the need for both caution and further research.

TPhP was introduced as an effective, low-toxicity alternative flame retardant. However, the U.S. EPA’s 2014 published report on BFR replacements lists moderate carcinogenic potential, high repeated dose risk, and very high risk for acute and chronic aquatic exposures for TPhP [16]. In fish models, the EPA report cites an acute LC50 of 0.4–0.85 mg/L in fish and a chronic LOEC (lowest-observed-effect concentration) of 0.037 mg/L for 30-day exposure in rainbow trout (*Oncorhynchus mykiss*). In 2023, TPhP was grouped with two other flame retardants, (tris(2-chloroethyl) phosphate (TCEP) and 4,4′-(1-methylethylidene)bis[2, 6-dibromophenol], also known as “tetrabromobisphenol A,” (TBBPA), to undergo Toxic Substances Control Act (TSCA) risk evaluation by the EPA [17]. Notably, TBBPA is the most widely used flame retardant worldwide and has been shown to impact the neurological system through endocrine dysregulation [18,19,20]. Increasing evidence of triphenyl phosphate’s toxicity has been found in zebrafish and multiple other related aquatic species [16,21,22,23]. Zebrafish (*Danio rerio*) are an effective model species for TPhP toxicity because the species and its development have already been widely studied, and since TPhP may particularly accumulate in aquatic vertebrates, its impacts on these species are critical to determine [7]. Zebrafish genetic and metabolic pathways are highly conserved with humans and other vertebrates, and as such these impacts are likely to be transferable to TPhP exposure in various vertebrate species and to humans [24].

High TPhP concentrations are clearly linked to teratogenicity in zebrafish and other aquatic organisms, with a 96 hpf LC50 for zebrafish reported as 5.3 mg/L [25]. Chronic larval TPhP exposure in zebrafish led to bioaccumulation in larvae and resulted in reduced locomotion, heart rate, and heart development at a concentration of 100 µg/L [22]. The same study also found several indicators of neurotoxicity, including reduced presence of several neurotransmitters, increased acetylcholinesterase activity, and a reduction in structural genes important for CNS development [22]. Zebrafish exposed to higher doses of TPhP (100 µg–5 mg/L) for prolonged periods also consistently display hepato- and cardiotoxicity, reduced length, and altered carbohydrate and lipid metabolism, among other altered metabolic pathways [21,26,27,28]. Nearly ubiquitous pericardial edema and impaired looping of heart chambers are reported at concentrations greater than or equal to 1 µM (0.33 mg/L) TPhP, resulting in the stunted “tube heart” phenotype associated with exposure to TCDD and retinoic acid and linked to the RXR pathway [27,29]. In both zebrafish cell lines and adult fish, similar doses of TPhP had considerable impacts on gene expression, altering pathways important for sexual development and lipid metabolism and increasing genetic markers for cardio- and hepatotoxicity and DNA repair [21,28,30,31,32].

There is much less conclusive data on the effects of lower TPhP concentrations comparable to those detected in the environment (0.01–8 µg/L), but some effects have been identified. Downregulation of structural genes important for the development of the nervous system was detected after exposure to concentrations as low as 4 µg/L [22]. Antiestrogenic effects of TPhP exposure have been investigated further in another small fish, the Japanese medaka (*Oryzias latipes*); chronic adult exposure increased the incidence of intersex and reduced female-chasing behavior in male fish, while female fish had smaller ovaries, reduced egg output, and lower transcription of the yolk precursor protein VTG [23,33]. The reproductive changes reported by these studies began at the lowest studied dose, 1.31 µg/L TPhP, offering evidence for developmental interference at environmentally plausible concentrations. However, the effects of very high chemical exposure levels are not always translatable to low doses. Given that the effects of TPhP on the heart are most widely studied at very high concentrations, it is important to determine whether these effects are detectable or relevant at environmental concentrations 50 to 1000 times lower than those used by many key studies characterizing the effects of TPhP on zebrafish. 

The goal of the present study is to determine the teratogenic effects of three environmentally relevant concentrations of TPhP (0.5, 1, and 5 µg/L; 1.5–15 nM). In this study, embryos were continuously exposed to TPhP from epiboly to 5 dpf, when the growth and heart development of the fish were measured and scored and molecular mechanisms of TPhP-induced pericardial edema, including *tbx5*, RXR, and the oxidative stress pathway, were examined. 

## 2. Materials and Methods

### 2.1. Chemicals

Triphenyl phosphate (TPhP, ID#, >99%), dimethyl sulfoxide (DMSO, ID#, >99.9%), and tricaine (ethyl 3-aminobenzoate methanesulfonate) were purchased from Sigma Aldrich (St. Louis, MO, USA). 

### 2.2. Zebrafish

All the animal research was approved by the Bates College Institutional Animal Care and Use Committee (IACUC) under animal welfare assurance number A3320-01. Wild-type zebrafish on the AB background were used in this study and were originally obtained from the Zebrafish International Research Center (ZIRC, Eugene, OR, USA). The adult animals were kept in an Aquaneering UV-filtered system under standard husbandry conditions, as described in Jönsson et al. 2007 [34]. Once collected, embryos were kept in 0.3× Danieau’s solution (17.40 mM NaCl, 0.21 mM KCl, 0.12 mM MgSO_4_∙7H_2_O, 0.18 mM Ca(NO_3_)_2_, and 1.5 mM HEPES). 

### 2.3. Chemical Exposure to TPhP

**Figure 1:**
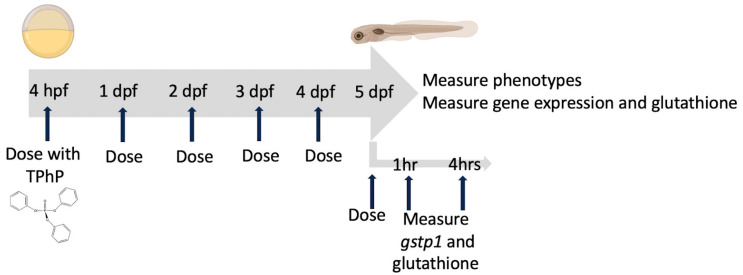
Experimental design and timing of measurements relative to TPhP dosing and development. The zebrafish images were created by BioRender.com.

Chemical exposure began at approximately 4 hpf; the embryos were confirmed to be fertilized and in epiboly before dosing. TPhP was prepared in DMSO and then diluted to concentrations of 0.5 µg/L, 1 µg/L, and 5 µg/L, resulting in a final concentration of 0.01% DMSO within all the TPhP treatment and DMSO control groups. A total of 60 embryos per exposure group replicate were raised in 250 mL beakers in 60 mL of dosing solution. For most measurements, the exposure period was 5 days, during which time the media was refreshed daily (Figure 1), and dead embryos were noted and removed. The one exception was for the measurement of the oxidative stress response where, because the response may be in recovery at the 24 h mark post-exposure [29], a shorter dosing and collecting interval was conducted, with 5 dpf embryos dosed at 5 µg/L for 1, 4, or 24 h before collection (Figure 1).

### 2.4. Phenotypic Assessment and Scoring

Larval growth and development were assessed at 5 days post fertilization. The larvae were photographed and measured on a Leica M165C microscope using Leica Application Suite X (LAS X) software (Leica Biosystems). Heart rate was measured manually as beats per 30 s for *n* = 15 fish per treatment, and two replicate measurements were averaged for each animal. The fish were immobilized with tricaine (75 mg/L final concentration) for all phenotypic evaluation except heart rate measurements. Pericardial edema severity was scored manually on a scale from 1 to 5 as follows: 5 = normal development, no pericardial space visible around heart, normal heart chamber morphology; 4 = mild edema, some pericardial space visible around heart, normal heart chamber morphology; 3 = moderate edema, pericardial area greater than heart area, normal heart chamber morphology; 2 = severe edema, edema leads to distortion or stretching of heart chambers, weak heartbeat; 1 = severe edema, stretching of heart chambers, edema of heart extends to body cavity or heartbeat is absent. These scoring methods were adapted from Panzica-Kelley et al. 2020, using a scoring matrix adapted from Sant et al. 2021 [35,36]. Pericardial area (including the atrium, ventricle, and pericardial space) and body length (head to the base of the tail, accounting for any spinal curvature) were also recorded for each animal.

To further determine if circulation was impacted by exposure to TPhP, a Nikon AX/AR inverted microscope was used to capture heart movement and circulation of 5 dpf control and chronically exposed (5 µg/L) larvae. 

### 2.5. RNA Extraction and qPCR

Quantitative real-time PCR (qPCR) was performed to assess the whole-larvae relative mRNA expression of genes potentially mediating TPhP toxicity. Three replicates containing 12 animals each were frozen at −20 °C for RNA extraction. Total RNA was extracted from each replicate using the Bio-Rad Aurum Total RNA Mini Kit (Bio-Rad, Billerica, MA, USA) according to manufacturer instructions. RNA quality and quantity were assessed by a NanoDrop One spectrophotometer (Thermo Fisher Scientific) prior to cDNA synthesis. cDNA was generated from 500 ng of template RNA using the iScript cDNA synthesis kit (Bio-Rad, Billerica, MA, USA). qPCR reactions of genes listed in Table 1 were performed in triplicate using SYBR Green Master Mix (Bio-Rad, Billerica, MA, USA) on an Agilent AriaMx Real-Time PCR system and data were analyzed by 2^–∆∆Ct^ method [37], with the reference gene beta-2-microglobulin (*b2m*) [38]. 

### 2.6. Measurement of Glutathione 

Glutathione was measured in the controls and TPhP-exposed 5 dpf larvae using the Cayman Chemical Glutathione Assay Kit (item no. 703002) following manufacturer instructions on a BioTek Synergy 2 Microplate Reader. Three replicates containing 15 animals each were dosed at 5 µg/L for 1, 4, or 24 h prior to measurement. To determine if there was a depletion of GSH upon exposure to TPhP, the ratio of GSH/GSSG was calculated [44].

### 2.7. Statistical Analysis

Statistical analysis of phenotypic data was performed using RStudio software Version 1.1. Body length and pericardial area data were both evaluated by Bartlett’s test to determine whether the variances differed between TPhP treatments; the variance in pericardial area was found to differ significantly by treatments, while the variance in body length did not. Accordingly, the pericardial area was analyzed by Welch’s ANOVA, while body length and glutathione expression data (*gstp1*) and ratios were analyzed by ANOVA followed by a Tukey’s HSD test. Ordinal data generated by phenotypic scoring was evaluated by a Kruskal–Wallis test with post hoc Dunn’s testing. An analysis of heart and RXR gene expression was performed in Prism using a Student’s T-test and multiple testing corrections to compare the control and TPhP-treated samples. A significance threshold of *p* = 0.05 was used for all the tests. Bar graphs, when shown, display the mean ± standard deviation.

## 3. Results

### 3.1. Effect of Environmental TPhP Concentrations on Heart Morphology

From epiboly to 5 dpf, the larvae exposed to the DMSO vehicle control or TPhP at 0.5, 1, or 5 µg/L were monitored for any abnormal development of the heart, particularly for pericardial edema and the distinctive TCDD-like tube heart effect previously reported for µM concentrations [29]. The larval pericardial area was measured at 5 days post fertilization, and edema was also qualitatively ranked in severity with 5 levels (see methods Section 2.4 for more details). Of all 191 fish phenotypically assessed, none displayed visibly altered or failed looping of the atrium and ventricle, a characteristic phenotype of TPhP exposure in the mg/L range.

**Figure 2:**
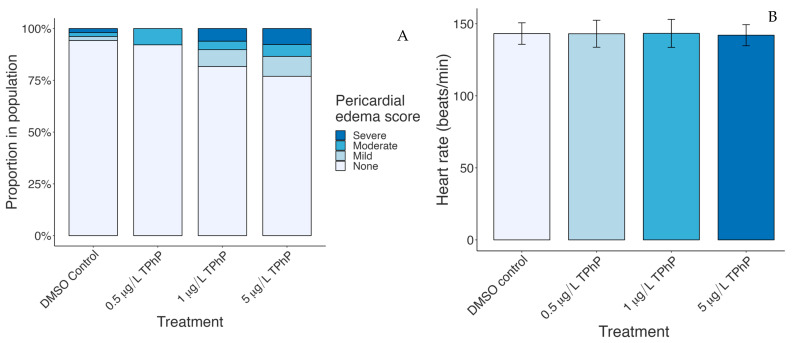
(**A**) Proportion of scored heart development phenotypes in fish treated with DMSO vehicle control or 0.5, 1, or 5 µg/L TPhP. Larvae were initially scored 5-1; scores of 1 and 2 are grouped and represented as “severe abnormality” on this chart. *n =* 52, 38, 49, 52 per group. (**B**) Heart rates of treated larvae. *n* = 15 per group.

The fish exposed to the DMSO vehicle displayed a baseline rate of 5.8% pericardial edema. While there was little change (7.7%) in the 0.5 µg/L TPhP exposure group, at both 1 and 5 µg/L TPhP the rate rose sharply to 17.3 and 23%, respectively (Figure 2). The score frequency within the sampled population is shown in Figure 2; scores of 1 and 2, which both indicate extreme pericardial edema, are combined for legibility. TPhP treatment led to a significant reduction in the median score of the TPhP treatment groups relative to the control (Kruskal–Wallis, *p* = 0.042) but a post hoc Dunn’s test did not reveal any single significant comparison relative to the control (*p* = 1.0, *p* = 0.44, *p* = 0.07 by treatment). However, there is a clear increase in rates of edema at the two higher doses, corresponding to an increased incidence of both mild and severe edema. A test of larval heart rate (*n =* 15 per treatment) revealed no significant difference between the control and the TPhP treated groups (Figure 2).

**Figure 3:**
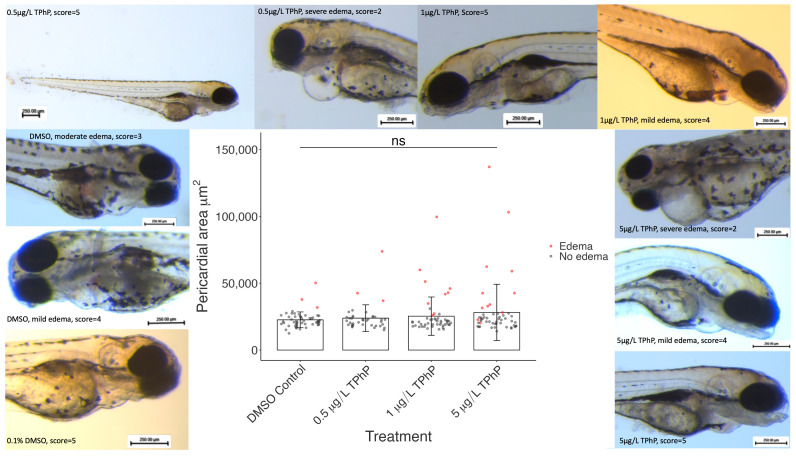
Pericardial areas of fish treated with DMSO vehicle control or 0.5, 1, or 5 µg/L TPhP. Data points are shown: black data points represent fish with an edema score of 5 (normal), and red points represent fish scored below 5 (i.e., displaying pericardial edema). Border: representative images of fish from all dosage groups with a range of heart development scores. *n =* 52, 28, 48, 52 per group, respectively.

In addition to an assigned score, the pericardial area of each fish was measured. There was no significant difference in the mean pericardial area between groups (*p* = 0.22), and no change in pericardial area was seen for fish that displayed normal phenotypic development (Figure 3). The pericardial areas of fish with edema (Figure 3, red data points) were greater overall in the TPhP treatment groups relative to the control fish, indicating that TPhP treatment may increase the severity of pericardial edema in addition to its prevalence.

The cardiac movement was also captured via video, and no gross differences between the control and experimental groups were found, although the blood supply to the heart appeared to be slightly slower in the TPhP treatment groups than in the control groups (Supplementary Materials Video S1).

### 3.2. Effect of TPhP on Larval Length

**Figure 4:**
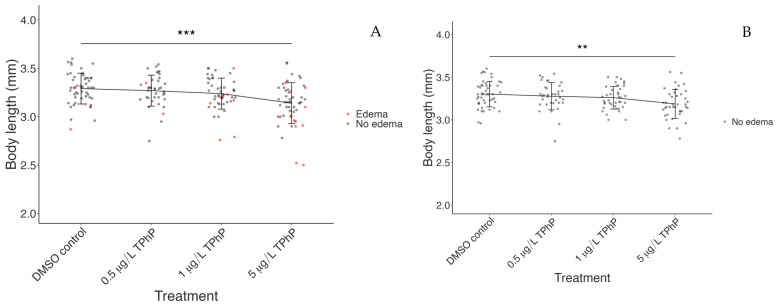
TPhP exposure significantly affected body length at 5 dpf relative to the DMSO vehicle control in both the whole population (**A**) and individuals without pericardial edema (**B**). Individual data points are shown; lines connect mean values for each treatment and bars show standard deviation. In (**A**) *n* = 52, 39, 48, and 52 per group; in (**B**) *n* = 49, 35, 40, and 40 per group. *p*-values: ** *p* < 0.01, *** *p* < 0.001.

In our study, the mean body length of larvae in the 5 µg/L treatment group was slightly, but very significantly, lower than the mean body length of the control (Figure 4A, control vs. 5 µg/L, *p* = 0.00012). The difference in mean body length between the control and 5 µg/L TPhP treatment groups was 0.14 mm (3.29 vs. 3.14 mm).

The fish with pericardial edema displayed significantly reduced body length overall compared to those with normal morphology (data not shown). Because edema potentially affects larval growth and development independent of TPhP’s other toxic effects, larval body length was also analyzed, with individuals displaying pericardial edema removed (Figure 4B). When analyzed this way, the mean body length of otherwise normally developing fish treated with 5 µg/L TPhP differed significantly from the mean body length of the normally developing control population (*p* = 0.0034).

### 3.3. Impacts of TPhP on RXR and Cardiac Gene Expression

**Figure 5:**
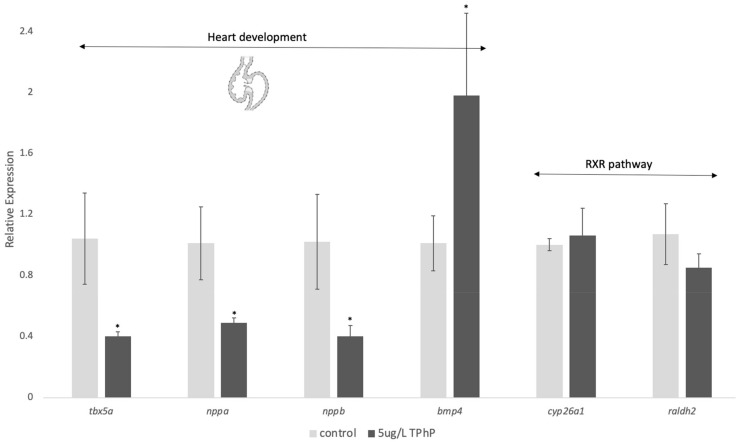
Expression of genes related to heart development and the RXR pathway in 5 dpf animals. Data are presented as the mean relative expression normalized to the housekeeping gene, *b2m*. Analysis of expression was performed in Prism using a Student’s *t*-test, adjusted for multiple testing, to compare the expression of a gene between the control and TPhP-treated samples, where an asterisk indicates a statistically significant change (*p* < 0.05) due to exposure.

Since overall heart morphology in the TPhP-treated fish was normal, we looked for gene expression changes in both edema-associated genes and canonical receptor targets of high TPhP concentrations. TPhP exposure had no significant effect on RNA levels of two marker genes for retinoic acid signaling and metabolism (Figure 5), *cyp6a1* and *raldh2,* involved in RA production and inactivation, respectively. A lack of evidence for interference in the RA signaling pathway led us to consider the potential role of a different heart development mechanism, the *tbx5a* pathway. At a concentration of 5 µg/L, *tbx5a* and its targets, *nppa* and *nppb,* were downregulated relative to the control, and *bmp4* was upregulated (Figure 5).

### 3.4. Effects of TPhP on Glutathione Metabolism and the Oxidative Stress Response

**Figure 6:**
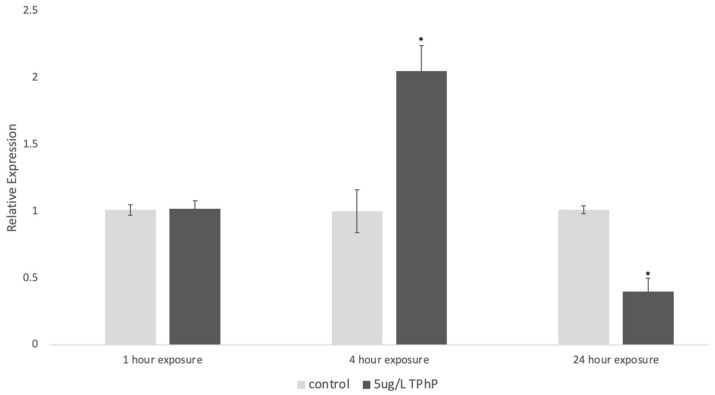
Expression of *gstp1* in 5 dpf animals dosed with TPhP for 1, 4, or 24 h prior to analysis of gene expression. Data are presented as the mean relative expression normalized to the housekeeping gene, *b2m*. Analysis of expression was performed in Prism using a two-way ANOVA with a Tukey’s HSD to compare the expression of the gene between the control and TPhP-treated samples, where an asterisk indicates a statistically significant change (*p* < 0.05) due to exposure and time.

**Figure 7:**
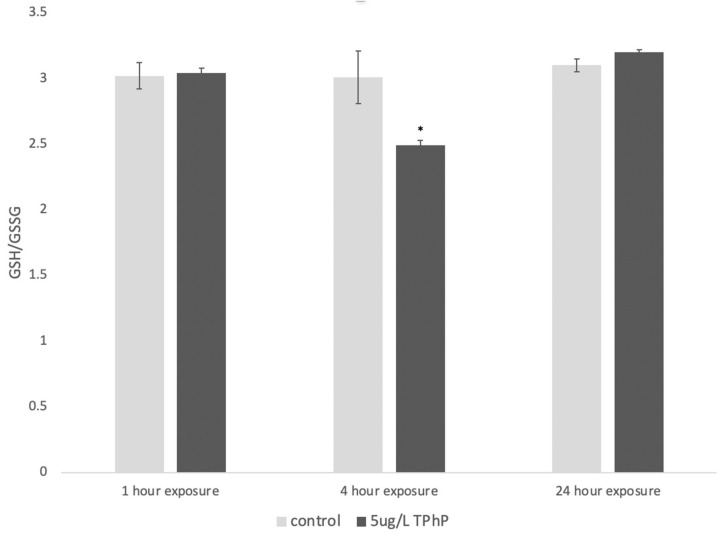
Ratio of reduced (GSH) to oxidized (GSSG) glutathione in 5 dpf embryos. Ratios are presented as an average with standard deviation, where an asterisk indicates a statistically significant change (*p* < 0.05) compared to all other values, as calculated by a two-way ANOVA with a Tukey’s HSD test.

Our data indicated that TPhP elicits an OSR at nM concentrations as measured by the expression of *gstp1* (Figure 6), a gene with high expression during zebrafish development [45], responsible for conjugating glutathione to electrophiles [46], and known to be upregulated in response to pro-oxidants [41,47,48]. The response was further confirmed by an increase in glutathione disulfide (GSSG) (Figure 7), the oxidized form of glutathione [49]. It is important to note that the timing of gene expression and glutathione measurements are key to understanding the OSR induced by exposure to TPhP. The OSR for organic compounds tends to peak soon after exposure [50], leaving the organism in “recovery” at the 24 h period post-exposure. A similar phenomenon was shown here, where *gstp1* and GSSG were increased shortly after exposure (at 4 h), but at 24 h, *gstp1* was downregulated, and GSSG was back to control levels (Figure 6 and Figure 7).

## 4. Discussion

While there have been many studies assessing the impact of TPhP exposure on zebrafish development, ours is one of the first to assess the phenotypic and molecular impacts of nM developmental exposures that represent environmentally relevant concentrations. At these concentrations, we observed some phenotypes that have been reported at uM concentrations (reduced length, pericardial edema), but cardiac development differed between the two concentrations in that no tube heart was observed at nM concentrations. The No observed effect level (NOEL) and the lowest observed adverse effect level (LOAEL) for developmental TPhP exposure differed based on the endpoint; the NOEL for heart rate and pericardial edema was 5 µg/L, whereas the LOAEL for body length and changes in targeted gene expression was 5 µg/L. As such, the molecular mechanisms underlying these effects were explored, and indeed, a novel pathway involving the *tbx5* transcription factor and downstream targets (*nppa*, *nppb*, and *bmp4*) was identified, whereas the RXR pathway showed no effect. We were also able to show that oxidative stress is induced by exposure to TPhP.

Reduced body length is also a well-known outcome of TPhP exposures in the nM to µM range [26,27,29,51]. This phenotype is likely linked to the upregulation of several miRNAs (miR-137 and 141) that subsequently downregulate genes involved in tail development [52]. Here, we replicate these findings at environmental TPhP concentrations, showing that chronic exposure to 5 µg/L TPhP significantly reduces body length relative to the control group and all lower treatments, and further that reduced body length at 5 µg/L is present independent of edema or an observable cardiotoxic phenotype. This indicates that the impacts of TPhP on whole-organism growth and development are not directly caused by changes to cardiac development and may not be mediated by the same pathway.

Cardiotoxicity, including the formation of a tube heart and pericardial edema, is a well-documented outcome of µM TPhP exposures [26,27,29,51,53,54]. Our data suggest that while TPhP is still a cardiotoxicant at low nM concentrations, the outcomes are not conserved (no tube hearts) or as stark (for edema data) as those seen at µM ranges. Cardiotoxicity is a known toxicological endpoint for flame retardants including other organophosphate flame retardants [53] and brominated flame retardants such as TBBPA [1].

However, our data is consistent with another study showing that, for concentrations in the µg/L range, edema was induced but heart rates were unchanged in larvae older than 60 hpf [25].

A complicating factor in the toxicology of TPhP is continued uncertainty around its exact mechanism of action. Despite this, TPhP is similar to other OPFRs in that it induces cardiotoxicity, hepatotoxicity, and altered lipid metabolism [1,28,30,32], but those outcomes do not seem to be driven by AHR [27]. Because TPhP has been shown to be involved in the retinoid X receptor (RXR) signaling pathway [25,26,29], we measured the expression of *cyp26a1* and *raldh2*, genes that are involved in RA inactivation and RA production, respectively [55,56]. Unlike the downregulation that has been reported for *cyp26a1* at uM exposure concentrations in zebrafish [25,26], at nM concentrations, there was no change to the expression of either gene (Figure 5). Because the mechanism by which TPhP interacts with the RXR pathway is unknown, it is hard to say why nM concentrations of TPhP do not repress the pathway in the same way as µM concentrations. It has been suggested that TPhP could be an RXR antagonist or act indirectly to decrease RXR-dependent pathways. Since only μM concentrations of RXR ligands have been studied in developing zebrafish, it is possible that nM concentrations of ligands do not alter the pathway and thus are not involved in mediating the TPhP toxicity we observed.

Given that overall heart function seemed to be intact in larvae following low-dose TPhP exposure, a molecular mechanism to explain increased pericardial area and edema in treated fish was sought. The transcription factor *tbx5* is a regulator of cardiac gene expression [57,58], including atrial natriuretic peptide (*nppa*) [43]. As a master regulator of cardiac gene expression, *tbx5* has been shown to be very sensitive to its own gene dosage, where too few (haploinsufficiency) or too many copies (gene duplication) lead to Holt–Oram syndrome, which is marked by congenital malformations of the heart [59]. While the regulation of tbx5 is still relatively unexplored, its regulation has been linked to a novel microRNA called miR-1218 [60]. Along with brain natriuretic peptide (*nppb*), *tbx5* and *nppa* are involved in regulating early heart development in both zebrafish and in medaka, where they are also important in circulation and osmoregulation [39,53,61]. The double mutation of *nppa*/*nppb* in zebrafish caused a similar edema phenotype to what we observed [39], resulting from altered expression of *tbx5* and the downstream gene *bmp4*, a protein involved in regulating the atrioventricular canal during heart development [62]. Similar phenotypes to our work (e.g., length and edema) and *tbx5a* pathway dysregulation have also been demonstrated in larval zebrafish exposed to high concentrations of glucose [43], and *tbx5a* loss of function in zebrafish is also known to impair heart development [63]. Interestingly, Du et al. (2015) investigated *tbx5a* and *bmp4* in relation to TPhP, but only measured their expression up to the protruding mouth stage (72 hpf) [53]. Early on, both genes were downregulated when animals were chronically exposed to 0.5 mg/L TPhP (1000× greater than our lowest dose); at 72 hpf, expression of *tbx5a* was unchanged but *bmp4* was upregulated, which is similar to our findings. These data suggest a role for the *tbx5a* cascade in the embryonic response to TPhP on which further work should be conducted.

The mechanism of action that links TPhP to edema has long been sought; a recent paper identified ioncyte abundance as one mediating factor [51]. Another parallel mechanism could involve the dysregulation of the redundant natriuretic peptides (*nppa*/*nppb*); their knockdown led to edema caused by increased extracellular matrix in the atrium [39]. In medaka, these peptides were linked to blood circulation and increased body fluid osmolality, perhaps another contributing factor to the edema and changes in circulation noted in our study [64].

Toxicity studies implicate TPhP in a wide and complex range of metabolic and developmental pathways, but one major source of its neurotoxic and developmental toxicity is believed to be the generation of reactive oxygen species (ROS) and interactions with oxidative stress defense systems [1,32,55,65,66,67,68]. A study of environmentally plausible TPhP concentrations in the fish *Labeo rohita* found significant increases in the levels of ROS production and lipid peroxidation following treatment, as well as an altered apoptotic profile—another indicator of oxidative stress [69]. Another study focused on TPhP and oxidative stress trends found a similar “recovery” effect on gene expression, where heme oxygenase 1 (*hmox1*), a protein that is sensitive to pro-oxidants [70], was downregulated in 96 hpf animals 24 h after TPhP exposure [65]. Because TPhP causes a change in the OSR within hours (Figure 6 and Figure 7), it is likely a “weak pro-oxidant” akin to tBHQ [50]. Mechanistically, it is likely to change the mitochondrial membrane potential in embryos, as reported by other studies [71,72]. This mechanism of action differs from chemicals like tert-Butyl hydroperoxide (tBOOH), which is a direct oxidant that upregulates the OSR within minutes [50]. Since TPhP does induce the OSR, its cardiotoxicity could be related to ROS-mediated cardiovascular apoptosis, which has been shown for diclofenac (an NSAID pain treatment) and TCDD [73,74,75], a potential cardiotoxic pathway that has not yet been explored in the context of TPhP. 

## 5. Conclusions

Our study showed that at low nM (environmentally relevant) concentrations of TPhP, the development of zebrafish was impacted in similar ways to previous studies at µM, including the loss of body length and pericardial edema. However, no tube heart formation was observed at these low concentrations, indicating that there may be differences in the mechanisms of action between µM and nM exposures. We found that the RXR pathway was not dysregulated, but that TPhP exposure altered the *tbx5a* cascade—an important cardiovascular development pathway—and invoked the oxidative stress response. Experiments to further elucidate the differences between nM and µM exposures at the molecular level are warranted, as well as investigation of a mechanism by which TPhP interacts with the *bmp4* pathway and represses *tbx5a* expression.

## Figures and Tables

**Table 1:** List of primers used for quantitative PCR.

Gene Name	Primer F (5’-3’)	Primer R (5’-3’)	Reference
*atrial natriuretic peptide (nppa/anf)*	ACAGCTCTGACAGCAACATGG	CTGATGCCTCTTCTGTTGCCA	[39]
*beta-2-microglobulin (b2m)*	GCCTTCACCCCAGAGAAAGG	GCGGTTGGGATTTACATGTTG	[38]
*bone morphogenetic protein 4 (bmp4)*	CGCAGCCCTAAACAAAGAG	TGATTGGTGGAGTTGAGATGAT	[40]
*brain natriuretic peptide (nppb/bnp)*	TGTTTCGGGAGCAAACTGGA	GTTCTTCTTGGGACCTGAGCG	[39]
*cytochrome P450 26a1 (cyp26a1)*	GATGCTCTGGAGCACTACATTC	GTTCTTGCTCGTCCGTCTTTAT	[26]
*glutathione S-transferase p1(gstp1)*	CGACTTGAAAGCCACCTGTGTC	CTGTCGTTTTTGCCATATGCAGC	[41]
*retinaldehyde dehydrogenase 2 (raldh2)*	GGGGTAAAGTGGTAAAACGC	GCAGTGGTCAAAAGCATGGC	[42]
*t-box transcription factor 5a (tbx5a)*	GGAATTTAAGGCCTCACGGTA	GATTTGCTGACGGCTGCATTCTGT	[43]

## Data Availability

Data is available upon request to LM Williams.

## Supplementary Materials

The following supporting information can be downloaded at: https://www.mdpi.com/article/10.3390/toxics12050368/s1; Video S1: Video of cardiac movement in 5 dpf control (A) and TPhP (B) larvae.
